# Southern Dental Association

**Published:** 1871-11

**Authors:** 


### SOUTHERN DENTAL ASSOCIATION.

During the meeting of the Southern Dental Association, held in Charleston, S. C., in April, 1871, the Committee on Memorials offered the following resolutions, which were unanimously adopted.

"The committee appointed to draft suitable resolutions in reference to the death of Dr. T. J. Crow, of Macon, Ga., and Dr. Warren Johnson, of Savannah, Ga., beg leave to offer the following:

WHEREAS, It has pleased Almighty God to take from us two of our beloved brethren; and

WHEREAS, It not only becomes us, but it is our mournful duty, to pay a worthy tribute to the memory of those who have been so long identified with us; therefore

*Resolved*, 1st. That in the death of our brethren, we recognize the hand of an Almighty Providence, and though with grief we mourn their loss, we bow with submission to the decree that has deprived us of those who won our highest esteem by their devotion to the interests of our profession, and who endeared themselves to us by their kindness of heart, which rendered them ever ready to alleviate the sufferings of humanity.

*Resolved*, 2nd. That we give expression to our fervent trust, that *our loss has been their gain*, inasmuch as they were ever known as faithful dentists, thorough gentlemen, and upright, patriotic citizens.

*Resolved*, 3d. That a page in the book of minutes of the Association, be dedicated to their memory.

*Resolved*, 4th. That a copy of these resolutions be forwarded to the several dental journals for publication; and, also, to the relatives of our departed brethren, with the assurance of our heartfelt sympathies in their bereavement.

(Signed.)

